# Sedation and Analgesia for Reduction of Pediatric Ileocolic Intussusception

**DOI:** 10.1001/jamanetworkopen.2023.17200

**Published:** 2023-06-07

**Authors:** Naveen Poonai, Daniel M. Cohen, Doug MacDowell, Rakesh D. Mistry, Santiago Mintegi, Simon Craig, Damian Roland, Michael Miller, Itai Shavit, and the Paediatric Emergency Research Networks (PERN) PAINT Study Group, Yvette Wang, Alan Nager, Theodore Heyming, Rebekah Burns, Indi Trehan, Matthew Lipshaw, Carmen Sulton, Joyce Li, Aderonke Ojo, Susan Kelly, Matthew Thornton, Kerry Caperell, Iluonose Amoni, Anna Abrams, Myto Duong, Muhammad Wassem, Adrienne Davis, Jocelyn Gravel, Evelyne Doyon Trottier, Neta Bar Am, Graham Thompson, Vikram Sabhaney, Garth Meckler, Rini Jain, Samina Ali, Silvia Bressan, Tiziana Zangardi, Giovanna Villa, Martina Giacalone, Michelle Seiler, Cyril Sahyoun, Fabrizio Romano, Zsolt Bognar, Szofia Hajosi-Kalcakosz, Lisa Amir, Said Hachimi-Idrissi, Zanda Pucuka, Astra Zviedre, Emīlija Zeltiņa, Natalie Phillips, Meredith Borland, Sharon O'Brien, Jeanette Marchant, Amit Kochar, Shane George, Victoria Pennington, Mark Lyttle, Jen Browning, Anna McLoughlin, Stuart Hartshorn, Chaman Urooj, Lucy Johnston, Emily Walton, Deepika Subrahmanyam Puthucode, Phil Peacock, James Conroy, Rafael Marañon, Silvia Garcia, Nuria Cahís, Amaia Cámara-Otegui, Arantxa Gomez, Maria Carbonero, Carlos Angelats-Romero, Adriana Yock-Corrales, Gabriela Hualde, Fabian Spigariol, Alex Donas, Cinthia Gübeli Linné, Alessia Rocchi, Alessia Pedrazzini, Giorgio Cozzi, Dino Barbi, Laura Baggio, Giovana La Fauci, Angela Mauro, Matthew Steimle, Danilo Buonsenso, Irma Ugalde, Gaby Nieva, Charlotte Harper, Idanna Sforzi, Shobhit Jain

**Affiliations:** 1 Departments of Pediatrics, Internal Medicine, Epidemiology and Biostatistics, Schulich School of Medicine and Dentistry, Western University, London, Ontario, Canada; 2 Department of Pediatrics, Nationwide Children’s Hospital, Columbus, Ohio; 3 Department of Pediatrics, University of Colorado School of Medicine, Aurora; 4 Pediatric Emergency Department, Biocruces Bizkaia Health Research Institute, Hospital Universitario Cruces University of the Basque Country, UPV/EHU. Bilbao, Basque Country, Spain; 5 Department of Paediatrics, Monash University, Clayton, Victoria, Australia; 6 SAPPHIRE Group, Health Sciences, Leicester University, Leicester, United Kingdom; 7 Paediatric Emergency Medicine Leicester Academic (PEMLA) Group, Children’s Emergency Department, Leicester Royal Infirmary, Leicester, United Kingdom; 8 Department of Paediatrics, Hadassah Hebrew University Hospitals, Jerusalem, Israel; 9 Rady Childrens Specialist, Encinitas, California; 10 Children's Hospital Los Angeles, Los Angeles, California; 11 Children's Hospital of Orange County, Orange, California; 12 Seattle Childrens Hospital, Seattle, Washington; 13 Cincinnati Children's Hospital Medical Center, Cincinnati, Ohio; 14 Children’s Healthcare of Atlanta, Atlanta, Georgia; 15 Boston Children's Hospital, Boston, Massachusetts; 16 Texas Children's Hospital, Houston; 17 Nemours/Alfred I. duPont Hospital for Children, New Castle County, Delaware; 18 State University of New York Upstate Medical University, Syracuse, New York; 19 Norton Children's Hospital, Louisville, Kentucky; 20 University of Minnesota Masonic Children's Hospital, Minneapolis; 21 Children's Hospital Colorado Anschutz Medical Campus, Aurora; 22 Southern Illinois University School of Medicine, Springfield; 23 Lincoln Medical Center, Bronx, New York; 24 The Hospital for Sick Children, Toronto, Ontario, Canada; 25 Centre Hospitalier Universitaire Sainte-Justine, Montreal, Quebec, Canada; 26 British Columbia Children's Hospital, Vancouver, British Columbia, Canada; 27 Children's Hospital of Eastern Ontario, Ottawa, Ontario, Canada; 28 Stollery Children's Hospital, Edmonton, Alberta, Canada; 29 University of Padova, Padova, Italy; 30 Gaslini Children's Hospital, Genova, Italy; 31 University Hospital Meyer, Florence, Italy; 32 Children's Hospital Zürich, Eleonore Foundation, Zürich, Switzerland; 33 Hôpitaux Universitaires de Genève, Geneva, Switzerland; 34 University Hospital Bern, Bern, Switzerland; 35 Heim Pál Children's Hospital, Budapest, Hungary; 36 Schneider Children's Medical Center, Petah Tikva, Iran; 37 Ghent University Hospital, Ghent, Belgium; 38 Children's Clinical University Hospital, Riga, Latvia; 39 Queensland Children's Hospital, South Brisbane, Australia; 40 Perth Children's Hospital, Nedlands, Australia; 41 Children’s Hospital at Westmead, Sydney, Australia; 42 Women's and Children's Hospital, North Adelaide, Australia; 43 Gold Coast University Hospital, Southport, Australia; 44 Sydney Children's Hospital, Randwick, Sydney, Australia; 45 Bristol Royal Hospital for Children, Bristol, England, United Kingdom; 46 Royal Hospital for Sick Children, Edinburgh, Scotland, United Kingdom; 47 Royal Hospital for Children, Glasgow, Scotland, United Kingdom; 48 Birmingham Children's Hospital, Birmingham, England, United Kingdom; 49 Evelina London Children's Hospital, London, England, United Kingdom; 50 Royal Alexandra Children's Hospital, Brighton, England, United Kingdom; 51 Leicester Royal Infirmary, Leicester, England, United Kingdom; 52 John Radcliffe Hospital, Oxford, England, United Kingdom; 53 Leeds General Infirmary, Leeds, England, United Kingdom; 54 Hospital Universitario Gregorio Marañon, Madrid, Spain; 55 Cruces University Hospital, Bilbao, Spain; 56 Consorci Corporació Sanitària Parc Taulí, Sabadell, Spain; 57 Hospital Donostia, San Sebastián, Spain; 58 Hospital Universitario Joan XXIII., Tarragona, Spain; 59 Hospital Virgen del Rocio, Sevilla, Spain; 60 Hospital Francesc de Borja, Valencia, Spain; 61 National Children's Hospital, San José, Costa Rica; 62 Hospital Garrahan, Buenos Aires, Argentina; 63 Reseau Hospitalier Neuchateloi, La Chaux-de-Fonds, France; 64 Children's Hospital, Lucerne Cantonal Hospital, Lucerne, France; 65 Children's Hospital of Eastern Switzerland St. Gallen, St. Gallen, Switzerland; 66 Policlinico of Milan, Milan, Italy; 67 Filippo Del Ponte Hospital, Varese, Italy; 68 Hospital Burlo Garofolo, Trieste, Italy; 69 Hospital for Women and Children, Verona, Italy; 70 Santobono-Pausilipon Children's Hospital, Naples, Italy; 71 Primary Children's Hospital, Salt Lake City, Utah; 72 Houston Medical Center, University of Texas, Houston; 73 Agostino Gemelli University Policlinic, Rome, Italy; 74 Children's Mercy Kansas City, Kansas City, Missouri

## Abstract

**Importance:**

Ileocolic intussusception is an important cause of intestinal obstruction in children. Reduction of ileocolic intussusception using air or fluid enema is the standard of care. This likely distressing procedure is usually performed without sedation or analgesia, but practice variation exists.

**Objective:**

To characterize the prevalence of opioid analgesia and sedation and assess their association with intestinal perforation and failed reduction.

**Design, Setting, and Participants:**

This cross-sectional study reviewed medical records of children aged 4 to 48 months with attempted reduction of ileocolic intussusception at 86 pediatric tertiary care institutions in 14 countries from January 2017 to December 2019. Of 3555 eligible medical records, 352 were excluded, and 3203 medical records were eligible. Data were analyzed in August 2022.

**Exposures:**

Reduction of ileocolic intussusception.

**Main outcomes and measures:**

The primary outcomes were opioid analgesia within 120 minutes of reduction based on the therapeutic window of IV morphine and sedation immediately before reduction of intussusception.

**Results:**

We included 3203 patients (median [IQR] age, 17 [9-27] months; 2054 of 3203 [64.1%] males). Opioid use was documented in 395 of 3134 patients (12.6%), sedation 334 of 3161 patients (10.6%), and opioids plus sedation in 178 of 3134 patients (5.7%). Perforation was uncommon and occurred in 13 of 3203 patients (0.4%). In the unadjusted analysis, opioids plus sedation (odds ratio [OR], 5.92; 95% CI, 1.28-27.42; *P* = .02) and a greater number of reduction attempts (OR, 1.48; 95% CI, 1.03-2.11; *P* = .03) were significantly associated with perforation. In the adjusted analysis, neither of these covariates remained significant. Reductions were successful in 2700 of 3184 attempts (84.8%). In the unadjusted analysis, younger age, no pain assessment at triage, opioids, longer duration of symptoms, hydrostatic enema, and gastrointestinal anomaly were significantly associated with failed reduction. In the adjusted analysis, only younger age (OR, 1.05 per month; 95% CI, 1.03-1.06 per month; *P* < .001), shorter duration of symptoms (OR, 0.96 per hour; 95% CI, 0.94-0.99 per hour; *P* = .002), and gastrointestinal anomaly (OR, 6.50; 95% CI, 2.04-20.64; *P* = .002) remained significant.

**Conclusions and Relevance:**

This cross-sectional study of pediatric ileocolic intussusception found that more than two-thirds of patients received neither analgesia nor sedation. Neither was associated with intestinal perforation or failed reduction, challenging the widespread practice of withholding analgesia and sedation for reduction of ileocolic intussusception in children.

## Key Points

**Question:**

What is the prevalence of opioid analgesia and sedation for reduction of pediatric ileocolic intussusception and what is their association with intestinal perforation and failed reduction?

**Findings:**

In this cross-sectional study of 3203 patients, use of opioids was documented in 12.6% of children, sedation in 10.6% of patients, and opioids plus sedation in 5.7% of children. Perforation and failure of reduction were rare outcomes in both children who received opioids and/or sedation and those who did not.

**Meaning:**

The findings of this study suggest that use of opioid analgesia and sedation are uncommon in children undergoing reduction of ileocolic intussusception.

## Teaser

This cross-sectional study characterizes the prevalence of opioid analgesia and sedation among children undergoing reduction of ileocolic intussusception and assesses their association with intestinal perforation and failed reduction.

## Introduction

Ileocolic intussusception refers to the invagination of the ileum through the ileocecal valve into the cecum.^1^ With a yearly incidence of approximately 56 of 100 000, ileocolic intussusception is an important cause of acute intestinal obstruction in children younger than 6 years.^2^ If untreated, intussusception can result in tissue ischemia, potentially leading to bowel necrosis, perforation, and shock.^3^ In countries with higher resources, case fatality is less than 1%,^1^ while it may be as high as 9% in nations with fewer resources.^3^ Transabdominal ultrasonography is the diagnostic modality of choice due to its high sensitivity (98%), safety, and availability.^4^ Emergent reduction of ileocolic intussusception using air or hydrostatic enema is the standard of care to prevent complications.^5^ Reduction involves inserting a French Foley catheter into the rectum and instilling water or air under pressure into the colon.^5^ No studies have objectively quantified pain during reduction, but it is believed to be painful based on analogies with colonoscopy, where the bowel is also distended with gas and children usually require sedation.^6^ In contrast, reduction of ileocolic intussusception is usually performed on awake children without sedation or analgesia. In the United States, only 7% of children with ileocolic intussusception receive sedation during reduction.^1^ The risk of bowel perforation during reduction is less than 1%.^5^

Although some studies report that sedation improves the success of intussusception reduction,^7,8,9,10^ most American and European radiologists do not support this practice.^11,12^ This may be due to requirements for specialized equipment, personnel skilled in airway maneuvers, or the belief that sedation may increase the risk of perforation during reduction.^13^ The management and complications of reduction of intussusception vary considerably by country,^1^ highlighting the importance of a global perspective of practice patterns to evaluate the risks of perforation during reduction and failed reduction related to sedation and analgesia. Our objective was to characterize practice patterns surrounding opioid analgesia and sedation for the reduction of ileocolic intussusception in children and to assess their association with intestinal perforation and failed reduction in an exploratory analysis.

## Methods

This study followed the Strengthening the Reporting of Observational Studies in Epidemiology (STROBE) reporting guideline. The institutional review board of each site approved the study and waived requirements for individual informed consent because patient data were deidentified.

### Design

We conducted a medical record review of children presenting to 86 emergency departments (EDs) in 14 countries within the Pediatric Emergency Research Networks (PERN) between January 1, 2017, to December 31, 2019 (Supplement 3). PERN is a global association of pediatric emergency care research networks, including the Pediatric Emergency Care Applied Research Network (PECARN) and the Pediatric Emergency Medicine Collaborative Research Committee (PEMCRC) of the American Academy of Pediatrics in the US, Pediatric Emergency Research Canada (PERC), Paediatric Research in Emergency Departments International Collaborative (PREDICT) in Australia and New Zealand, Paediatric Emergency Research in the UK and Ireland (PERUKI), Research in European Pediatric Emergency Medicine (REPEM), Research Network of the Spanish Society of Pediatric Emergency/Spanish Pediatric Emergency Medicine Research Group (RISEUP/SPERG), and Network for Research and Development of Pediatric Emergency Medicine in Latin America (Red de Investigacion y Desarrollo de la Emergencia Pediatrica Latinoamericana; RIDEPLA). Together, PERN research networks have access to data from more than 5 million pediatric ED presentations annually and more than 200 hospitals.

### Eligibility

We included patients aged 4 to 48 months with a sonographic diagnosis of ileocolic intussusception who underwent attempted reduction of intussusception. Medical records were consecutively identified through electronic queries at each institution. We excluded repeat presentations of intussusception.

### Data Collection

Data were deidentified before being entered into an electronic case report form hosted on Research Electronic Data Capture (REDCap)^14^ by a research assistant or coinvestigator at each site. One coinvestigator (D.M.) reviewed the data for accuracy. We collected demographics, symptoms, pain scores, analgesics and sedative medications, time to reduction, reduction parameters (pneumatic or hydrostatic reduction, insufflation pressure, personnel performing the procedure and training level, number of attempts, and success of reduction), sedation- and reduction-related adverse events, and operative management. Source documents included the medical record, anesthetic record, radiology, and operative reports. We defined analgesia as any pharmacologic nonsedative agent administered in the ED for pain management within 120 minutes prior to reduction (eg, ibuprofen, acetaminophen, opioids). We defined sedation as any pharmacologic agent with anxiolytic, sedative, dissociative, or anesthetic properties administered immediately prior to reduction (eg, ketamine, midazolam). Although some opioids (eg, morphine) have sedating properties, we elected to classify these as analgesics because we believed they were primarily used for this purpose. Time to reduction was measured from triage assessment to radiology department entry. A failed reduction was defined as all enema attempts failing to reduce intussusception as documented by the physician performing the procedure. Successful reduction was defined as such regardless of the number of enema attempts as documented by the physician performing the procedure. Adverse events were based on the Medical Dictionary of Regulatory Activities,^15^ and recorded at any time during sedation or reduction from the physician or nursing anesthetic record.

### Outcomes

The primary outcomes were opioid analgesia within 120 minutes of reduction based on the therapeutic window of IV morphine^16^ and sedation immediately before reduction of intussusception. Exploratory outcomes included associations between opioid analgesia and sedation and intestinal perforation during reduction and failed reduction.

### Sample Size Considerations

Based on an expected incidence of intussusception in children (56 of 100 000),^2^ the risk of perforation during reduction of 0.6%,^17^ and the risk of failed reduction of 13.1%,^17^ we believed our study period would capture 3000 to 3500 patient encounters and yield a sample size supporting a multivariable model with at most 2 factors for perforation and at most 32 factors for failed reduction in keeping with a general requirement of 10 to 12 covariates per outcome.^18^

### Statistical Analysis

Demographic characteristics were summarized with counts and percentages for categorical data and means and SDs or medians and interquartile ranges (IQRs) for continuous data. Bivariate and separate multivariable analyses were used to explore the associations between (1) perforation and the following prespecified covariates: age, sex, analgesia (opioids with or without nonopioid analgesics; nonopioid analgesics alone) at triage and within 120 minutes of reduction, triage pain assessment, sedation for reduction, opioids with or without nonopioid analgesics plus sedation, duration of symptoms prior to reduction, and number of reduction attempts and (2) failed enema reduction and the following prespecified covariates: age, sex, analgesia (opioids with or without nonopioid analgesics; nonopioid analgesics alone) at triage and within 120 minutes of reduction, sedation for reduction, opioids with or without nonopioid analgesics plus sedation, duration of symptoms prior to reduction, type of enema reduction (pneumatic vs hydrostatic), and preexisting gastrointestinal anomaly. Fentanyl was excluded from the opioid category for bivariate and multivariable modeling because we could not reliably ascertain that it was administered within its 60-minute therapeutic window. We included covariates in adjusted (multivariable) models if they were biologically plausible and had an unadjusted association with the outcome significant at a threshold of *P* < .05. Unadjusted bivariate and adjusted multivariable odds ratios (ORs) and 95% CIs were obtained from generalized mixed-effects logistic regression models with the factors of interest as fixed effects and site as a random effect using a simple variance components covariance structure with random intercept. Analyses were conducted using SPSS version 27 (IBM SPSS). A type I error rate of 0.05 was used to reject the null hypothesis of no association. All *P* values were 2-tailed. Data were analyzed using complete case analysis, and the analysis took place in August 2022.

## Results

### Participants

Overall, 3555 records were screened, and 3203 were eligible and included in the analysis (2054 [64.1%] males; median [IQR] age, 17 [9-27] months) (Figure). Most patients were from the US (1710 [53.4%]), followed by Canada (421 [13.1%]), Italy (229 [7.1%], Australia (221 [6.9%]), and the UK (148 [4.6%]) (eAppendix 1 in Supplement 1). The most common presenting symptoms in patients were abdominal pain (2283 of 3187 [71.6%]) and vomiting (2184 of 3187 [67.2%]). Seven patients had a pathologic lead point (Table 1). Preexisting gastrointestinal anomalies were documented in 32 of 3203 (1.0%) patients (Table 2).

**Figure.**
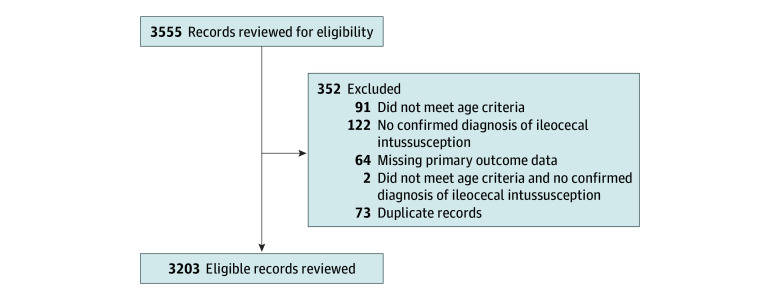
Flow Diagram of Medical Records Reviewed

**Table 1.** Demographic Characteristics and Clinical Characteristics of Children Diagnosed With Ileocolic Intussusception

Characteristic	Patients, No. (%), n = 3203
Age, median (IQR), mo	17 (9-27)
Sex, n = 3203	
Male	2054 (64.1)
Female	1149 (35.9)
Clinical presentation, n = 3187^a^	
Abdominal pain	2283 (71.6)
Vomiting	2184 (67.2)
Poor feeding	841 (26.4)
Irritability, fussiness, or episodic crying	854 (26.8)
Bloody stools^a^	792 (21.8)
Lethargy or fatigue	549 (17.2)
Fever	416 (13.0)
Diarrhea	470 (14.7)
Pallor	291 (9.2)
Nausea	177 (5.6)
Constipation	50 (1.6)
Syncope or altered level of consciousness	24 (0.8)
Dehydration or decreased urine output	26 (0.8)
Upper respiratory tract symptoms	24 (0.8)
Abdominal distension	11 (0.3)
No symptoms documented	7 (0.2)
Other^b^	46 (1.4)
Duration of symptoms prior to ED arrival, median (IQR), h, n = 2580	24 (9, 48)
Pathologic lead point, n = 3203^c^	7 (0.2)

Abbreviation: ED, emergency department.

^a^ Some patients had more than 1 symptom recorded, and there was no documentation of symptoms in 16 patients.

^b^ Stool described as black or red.

^c^ These included Meckel diverticulum (n = 5), lymph node (n = 1), and terminal ileum duplication cyst (n = 1).

**Table 2.** Preexisting Gastrointestinal Anomalies

Anomaly	Patients, No. (%), n = 3203
Meckel diverticulum	13 (0.4)
Henoch-Schoenlein purpura	6 (0.2)
Colonic lympho-nodular hyperplasia and ileocecal valve protrusion	2 (0.1)
Umbilical hernia	2 (0.1)
Crohn disease	1 (0)
Congenital imperforate anus and full repair with colostomy takedown	1 (0)
Congenital tracheoesophageal fistula, anorectal malformation	1 (0)
Cystic fibrosis	1 (0)
Duodenal bulb ulcer and gastrostomy tube	1 (0)
Previous intussusception and x-linked lymphoproliferative disorder	1 (0)
Previous intussusception, duodenal atresia repair, malrotation	1 (0)
Juvenile polyposis	1 (0)
Vertebral defects, anal atresia, cardiac defects, tracheo-esophageal fistula, renal anomalies, and limb abnormalities	1 (0)

### Pain Assessment and Analgesia at Triage

Pain assessment was documented in 1859 of 3112 patients (59.7%). The most common instruments were the Faces, Legs, Activity, Cry, Consolability (FLACC) scale (1530 of 1859 [82.3%]; median [IQR] score, 0 [0-2]) and the Faces Pain Scale—Revised (FPS-R) (127 of 1859 [6.8%]; median [IQR] score, 1 [0-5]). Pharmacologic analgesia was documented in 305 of 3171 patients (9.6%), most commonly acetaminophen (181 of 305 [59.3%]).

### Sedation and Analgesia Within 120 Minutes of Reduction of Intussusception

Any analgesia was documented in 466 of 3175 patients (14.7%), and morphine was the most common analgesic used (276 of 466 [59.2%]) (Table 3 and Table 4). Opioids were documented in 395 of 3134 patients (12.6%). Sedation was documented in 334 of 3161 patients (10.6%), and midazolam was the most common sedative used (168 of 334 [50.3%]) (Tables 3 and 4). Opioids plus sedation was documented in 178 3134 patients (5.7%).

**Table 3.** Unadjusted and Adjusted Analyses of Variables Associated With Perforation During Reduction of Ileocolic Intussusception

Variable	Patients, No. (%)	Perforation (%)		Unadjusted	*P* value^b^	Adjusted	*P* value
		Yes (n = 13)	No (n = 3190)	OR (95% CI)^a^		OR (95% CI)	
Demographics							
Age, median (IQR), mo, n = 3203		8 (6-13)	17 (9-27)	0.93 (0.87-1.01)	.07	NA	NA
Sex, n = 3203							
Female	1149 (35.9)	6 (0.5)	1143 (99.5)	1 [Reference]	.44	NA	NA
Male	2054 (64.1)	7 (0.3)	2047 (99.7)	0.65 (0.22-1.94)		NA	
Pain assessment at triage, n = 3112							
No	1253 (40.3)	6 (0.5)	1247 (99.5)	1 [Reference]	.67	NA	NA
Yes	1859 (59.7)	7 (0.4)	1852 (99.6)	0.79 (0.26-2.34)		NA	
Opioids with or without nonopioid analgesia at triage, n = 3171							
No	3120 (98.4)	13 (0.4)	3107 (99.6)	1 [Reference]	.99	NA	NA
Yes	51 (1.6)	0	51 (100)	0		NA	
Nonopioid at triage, n = 3171							
No	2919 (92.1)	12 (0.4)	2907 (99.6)	1 [Reference]	.97	NA	NA
Yes	252 (7.9)	1 (0.4)	251 (99.6)	0.97 (0.13-7.45)		NA	
Opioids with or without nonopioid analgesia within 120 min of reduction, n = 2975^c^							
No	2663 (89.5)	10 (0.4)	2653 (99.6)	1 [Reference]	.15	NA	NA
Yes	312 (10.5)	3 (1.0)	309 (99.0)	2.58 (0.71-9.41)		NA	
Nonopioid analgesia within 120 min of reduction, n = 2602							
No	2490 (95.7)	7 (0.3)	2483 (99.7)	1 [Reference]	.99	NA	NA
Yes	112 (4.3)	0	112 (100.0)	0		NA	
Nonopioid analgesia within 60 min of reduction, n = 2528							
No	2490 (98.5)	7 (0.3)	2483 (99.7)	1 [Reference]	.99	NA	NA
Yes	38 (1.5)	0	38 (100.0)	0		NA	
Sedation for reduction, n = 3161^d^							
No	2827 (89.4)	12 (0.4)	2815 (99.6)	1 [Reference]	.74	NA	NA
Yes	334 (10.6)	1 (0.3)	333 (99.7)	0.70 (0.09-5.44)		NA	
Opioids with or without nonopioid analgesia within 120 min plus sedation, n = 3134							
No	2956 (94.3)	10 (0.3)	2946 (99.7)	1 [Reference]	.02	1 [Reference]	.56
Yes	178 (5.7)	3 (1.7)	175 (98.3)	5.92 (1.28-27.42)		1.29 (0.55-3.00)	
Median (IQR) duration of symptoms prior to reduction in hours, n = 2460	NA	2.83 (1.44-3.95)	2.92 (1.77-4.48)	0.96 (0.77-1.18)	.68	NA	
Median (IQR) No. of reduction attempts, n = 2397	NA	1.5 (1-3)	1 (1-1)	1.48 (1.03-2.11)	.03	0.95 (0.77-1.17)	*P* < .62

Abbreviations: NA, not applicable; OR, odds ratio.

^a^ Odds ratios and 95% CIs compare the odds of perforation for each group vs the reference category, an increase in age by 1 month, an increase in number of reduction attempts by 1 attempt, or an increase in time to reduction by 1 hour.

^b^ *P* values test for a difference between any levels of a categorical factor, or for an odds ratio of zero for a continuous factor.

^c^ Analgesia data are available in eTable 1 of Supplement 1.

^d^ Sedation for reduction data are available in eTable 2 of Supplement 1.

**Table 4.** Unadjusted and Adjusted Analyses of Variables Associated With Failed Reduction of Ileocolic Intussusception^a^

Variables	Patient, No. (%)	Failed reduction (%)		Unadjusted OR (95% CI)^b^	*P* value^c^	Adjusted OR (95% CI)^b^	*P* value^c^
		Yes, n = 484	No, n = 2700				
Demographics							
Age, median (IQR), mo, n = 2953		10 (7-20)	18 (9-28)	0.96 (0.95-0.97)	<.001	1.05 (1.03-1.06)	<.001
Sex, n = 3184							
Female	1146	161 (14.0)	985 (86.0)	1 [Reference]	.18	NA	NA
Male	2038	323 (15.8)	1715 (84.2)	1.15 (0.94-1.41)			
**Triage**							
Pain assessment at triage, n = 3095							
No	1246	221 (17.7)	1025 (82.3)	1 [Reference]	.002	NA	NA
Yes	1849	253 (13.7)	1596 (86.3)	0.74 (0.60-0.90)			
Opioids with or without nonopioids analgesia at triage, n = 3155							
No	3104	465 (15.0)	2639 (85.0)	1 [Reference]	.09	NA	NA
Yes	51	12 (23.5)	39 (76.5)	1.75 (0.91-3.36)			
Nonopioid analgesia at triage, n = 3155							
No	2905	447 (15.4)	2458 (84.6)	1 [Reference]	.15	NA	NA
Yes	250	30 (12.0)	220 (88.0)	0.75 (0.51-1.11)			
**Analgesia**							
Opioids with or without nonopioid analgesia within 120 min of reduction, n = 2972^d^							
No	2661	279 (14.2)	2282 (85.8)	1 [Reference]	<.001	1 [Reference]	.09
Yes	311	68 (21.9)	243 (78.1)	1.69 (1.26-2.25)		1.44 (0.94-2.22)	
Nonopioid analgesia within 120 min of reduction, n = 2599							
No	2488	2346 (13.9)	2142 (86.1)	1 [Reference]	.68	NA	NA
Yes	111	17 (5.3)	94 (84.7)	1.12 (0.66-1.90)		NA	
Nonopioid analgesia within 60 min of reduction, n = 2525							
No	2488	346 (13.9)	2142 (86.1)	1 [Reference]	.59	NA	NA
Yes	37	4 (10.8)	33 (89.2)	0.75 (0.26-2.13)			
**Sedation**							
Sedation for reduction, n = 3157^e^							
No	2824	425 (15.0)	2399 (85.0)	1 [Reference]	.68	NA	NA
Yes	333	53 (15.9)	280 (84.1)	1.07 (0.78-1.46)			
Opioids with or without nonopioid analgesia within 120 min plus sedation, n = 2919							
No	2882	416 (14.7)	2406 (85.3)	1 [Reference]	.19	NA	NA
Yes	97	19 (19.6)	78 (80.4)	1.41 (0.84-2.35)			
Median (IQR) duration of symptoms prior to reduction in hours	2459	3.07 (1.92-5.27)	2.87 (1.73-4.42)	1.04 (1.02-1.07)	<.001	0.96 (0.94-0.99)	.002
Type of enema reduction, n = 3181							
Air	2370	325 (13.7)	2045 (86.3)	1 [Reference]	<.001	1 [Reference]	.59
Hydrostatic	811	157 (19.4)	654 (80.6)	1.51 (1.23-1.86)		1.11 (0.76-1.61)	
Preexisting gastrointestinal anomaly, n = 3178^f^							
No	3146	469 (14.9)	2677 (85.1)	1 [Reference]	<.001	1 [Reference]	.002
Yes	32	12 (37.5)	20 (63.5)	3.43 (1.66-7.05)		6.50 (2.04-20.64)	

Abbreviation: OR, odds ratio.

^a^ Failed reduction (yes/no) was not documented in 19 encounters.

^b^ Odds ratios and 95% CIs compare the odds of failed reduction for each group vs the reference category, an increase in age by 1 month, an increase in number of reduction attempts by 1 attempt, or an increase in time to reduction by each 1-hour increment.

^c^ *P* values test for a difference between any levels of a categorical factor, or for an odds ratio of zero for a continuous factor.

^d^ Analgesia data are available in eTable 1 of Supplement 1.

^e^ Sedation for reduction data are available in eTable 2 of Supplement 1.

^f^ Types of anomalies are listed in Table 2.

### Intestinal Perforation During Reduction

There were 13 of 3203 cases (0.4%) of perforation. Among these, 10 (77%) were diagnosed radiologically and 3 (23%) were diagnosed clinically. The median (IQR) age was 8 (6.5-12) months, insufflation pressure was 120 (120-120) mmHg, and time from triage to reduction was 140 (75.5-186.8) minutes. None had preexisting gastrointestinal anomalies or a pathologic lead point. Six of 13 patients (46 %) underwent more than 1 reduction attempt. In the unadjusted analysis, only opioids with or without nonopioid analgesics within 120 minutes of reduction plus sedation (OR, 5.92; 95% CI, 1.28-27.42; *P* = .02) and more reduction attempts were significantly associated with intestinal perforation (OR, 1.48; 95% CI, 1.03-2.11; *P* = .03). In the adjusted analysis, neither covariate remained significant (Table 3).

### Reduction of Intussusception

Data on reduction method was reported in 3184 patients. Reductions were attempted mostly using air enema (2372 of 3184 patients [74.5%]) and were successful in 2700 of 3184 attempts (84.8%). Reduction was primarily performed by radiologists in 3030 3157 patients (96.0%); specifically, the consultant radiologist in 2299 of 3157 patients (72.8%). Five of 7 patients (71%) with a pathologic lead point had a failed reduction. In the unadjusted analysis, younger age, lack of triage pain assessment, opioids with and without nonopioid analgesics within 120 minutes of reduction, longer duration of symptoms, hydrostatic enema reduction, and preexisting gastrointestinal anomaly were significantly associated with failed reduction. In the adjusted analysis, only older age (OR, 1.05 per month; 95% CI, 1.03-1.06; *P* < .001), shorter duration of symptoms (OR, 0.96 per hour; 95% CI, 0.94-0.99; *P* = .002), and preexisting gastrointestinal anomaly remained significant (OR, 6.50; 95% CI, 2.04-20.64; *P* = .002) (Table 4).

### Adverse Events

Death occurred in 1 of 3203 patients (0.03%), an 11-month-old male with a preexisting cardiomyopathy, who had several unsuccessful reduction attempts with hydrostatic enema that resulted in perforation of the transverse colon, leading to cardiopulmonary arrest. This patient received neither analgesia nor sedation. An adverse event related to sedation or analgesia was documented in 48 of 548 patients (8.7%), most commonly a decrease in oxygen saturation below 92% (10 of 48 patients [20.8%]). Nonfatal adverse events related to reduction of intussusception were recorded in 59 of 3166 patients (1.8%). These included vomiting (31 [1.0%]), intestinal perforation (13 [0.4%]), transient hypoxia (13 [0.4%]), transient bradycardia (3 [0.09%]), and transient apnea (2 [0.06%]).

## Discussion

In this international cross-sectional study, more than two-thirds of children received neither analgesia nor sedation for the reduction of ileocolic intussusception. However, neither analgesia, sedation, nor their combination was associated with intestinal perforation or failed reduction. In fact, the strongest covariate associated with failed reduction was preexisting gastrointestinal anomaly. Our results suggest the infrequency of provision of analgesia and sedation for this likely painful procedure and challenge the widespread practice of withholding analgesia and sedation.

Our perforation rate was consistent with a published rate of less than 1%,^5^ and our results are corroborated by a recent systematic review^17^ of 849 propofol-based sedations for reduction of intussusception in children where the incidence of intestinal perforation was 0.6%. Current reluctance to sedate children for reduction of intussusception appears to be common in the US^11^ and Europe,^12^ the source of most of our data and may stem from a study involving a porcine model where an induced Valsalva maneuver was deemed protective through decreasing transmural pressure with associated lower risk of perforation.^13^ Due to the small number of perforations, we were unable to adjust for risk factors, such as high insufflation pressures (≥120 mm Hg),^13^ duration of symptoms for more than 12 hours,^19^ lack of a Valsalva maneuver,^13^ dehydration,^19^ and younger age.^9^ Adverse events were uncommon, but importantly, our study was not powered to detect all adverse events, and it was not possible to determine their exact cause, whether related to sedation, analgesia, or procedure. Nevertheless, we found that neither opioid analgesia nor sedation within 2 hours of reduction was independently associated with perforation, in concert with the findings of Yeoh et al,^20^ where opioid analgesia within 2 hours of reduction was provided to 65.8% of Australian children and no perforations were reported.

Only 9.6% and 14.9% of patients received analgesia at triage and within 120 minutes of reduction, respectively. Although abdominal pain is a frequent presenting feature of intussusception in children,^21^ the most likely explanation for not providing analgesia at triage was low pain scores. Fortunately, pain assessment was most often performed using the FLACC scale, an appropriate behavioral instrument validated in children younger than 5 years.^22^ However, less than 60% of patients had a documented pain assessment. Along with being mandated by The Joint Commission,^23^ the importance of consistent pain assessment using age-appropriate instruments cannot be overemphasized as these have been associated with a greater likelihood of receiving analgesia in the ED.^22^

Several features of intussusception predispose to suboptimal pain management. First, intussusception primarily affects children younger than 5,^1^ and there is evidence that young children are less likely to receive analgesia than their adolescent counterparts.^24,25^ The reasons are multifactorial and include clinician uncertainty with medication dosing, concerns surrounding adverse effects, and the inability of young children to verbalize their needs.^25^ Second, children with abdominal pain (as opposed to musculoskeletal pain) are less likely to receive analgesia in the ED.^24^ This may be grounded in a historical misconception that analgesia may mask the signs of a surgical indication.^26^ Third, reduction often occurs in a radiology suite, where health care clinicians skilled in the assessment and management of pain and sedation are not always readily available. Fourth, the infrequency of sedation and analgesia for children with intussusception may also reflect the paucity of literature to inform clinical guidelines and medical directives. Although the American Academy of Pediatrics has recommended using physical, psychological, and pharmacological strategies to reduce pain and distress for children undergoing diagnostic and therapeutic procedures, there is no specific mention of the reduction of intussusception.^27^ Moreover, intussusception-specific guidelines from the United Kingdom do not mention analgesia. There have also been no published evaluations of the severity of pain and distress during intussusception. Interestingly, our lower rate of analgesia contrasted with the Yeoh et al^20^ sample where 61 of 73 children (83.5%) were administered analgesia prior to reduction. Australian practice patterns may reflect the development of hospital guidelines recommending analgesia as part of general management.^28^

The frequency of failed reduction in our sample (15.2%) is consistent with previous reports.^19^ The findings of a recent systematic review that included 1434 children who underwent sedation reported a success rate of 86.9% under sedation.^17^ Our finding that neither analgesia nor sedation was associated with failed reduction is in line with evidence that sedation actually improves the success rate of both hydrostatic^7,8,10^ and pneumatic enema reduction.^9,29^ For reasons that remain uncertain, in the adjusted analysis, we found that older age and shorter duration of symptoms were associated with an increased odds of failed reduction. Importantly, the strength of these associations was very weak and future prospective studies should control for factors, such as clinician experience, length of intussusception, and duration of symptoms.

Our findings provide preliminary evidence that neither opioid analgesia nor sedation is associated with perforation or failed reduction. Although this may inform the decision to consider analgesia and sedation, there are no studies that have rigorously characterized the pain and distress associated with reduction of intussusception. Clinicians must also weigh the potential benefit of sedation for patient comfort with the need for specialized equipment, monitoring, and personnel trained in pediatric resuscitative maneuvers outside the acute care setting. Nevertheless, our work lays the foundation for future interventional studies that may provide definitive evidence of distress during reduction of intussusception and evaluate the risks and benefits of providing analgesia and sedation, while controlling for risk factors for perforation and failed reduction, such as age and clinician experience.

### Limitations

This study had limitations. It is, to our knowledge, the largest study to date on this topic and provides a global perspective of children undergoing reduction of ileocolic intussusception who received sedation or opioid analgesia. Participating sites were primarily academic centers, where most reductions of intussusception in children are performed, and our results are likely only generalizable to these institutions. Due to the retrospective nature of the data, we were unable to characterize factors that may have influenced the provision of opioids and sedation, such as the timing of prehospital analgesia, inability to tolerate oral medication, and specific contraindications. We did not collect data on nonpharmacologic strategies, such as distraction that may have been facilitated by a caregiver or child life specialist. We were unable to characterize other possible risk factors for perforation and failed reduction, including degree of behavioral resistance during reduction, dehydration, duration of symptoms prior to reduction, length of intussusception, and clinician experience. This may have reduced the precision of our ORs. Pain during reduction may have been an important factor of failed reduction and perforation. This data was not available and highlights the importance of evaluating distress during reduction of intussusception. Most importantly, our results were highly dependent upon, and therefore limited to, the accuracy and completeness of the information contained in the medical record. Given that this was an unfunded global study, 2 blinded data abstractors at each site were not possible.

## Conclusions

The findings of this multinational cross-sectional study suggest that reduction of pediatric ileocolic intussusception can be successfully performed in most children with a very low risk of perforation. More than two-thirds of patients received neither sedation nor analgesia within 120 minutes of reduction. Our findings challenge the widespread practice of withholding analgesia and sedation and lay the foundation for future prospective studies exploring the benefits of sedation or analgesia for reduction of intussusception in children.

## Notes

Supplement 1. **eAppendix.** PAINT Study Group **eTable 1.** Analgesics and Sedatives to Accompany Table 3 **eTable 2.** Analgesics and Sedatives to Accompany Table 4

Supplement 2.
Nonauthor Collaborators

Supplement 3.
Data Sharing Statement
