# Errors in Byline

**DOI:** 10.1001/jamanetworkopen.2024.14085

**Published:** 2024-05-01

In the Original Investigation titled “Sedation and Analgesia for Reduction of Pediatric Ileocolic Intussusception,”^1^ published June 7, 2023, names of group authors were inadvertently omitted from the published article. This article has been corrected.^1^
