# Supplementary material for: Sedation and Analgesia for Reduction of Pediatric Ileocolic Intussusception
Source: JAMA Netw Open. 2023 Jun 7;6(6):e2317200. doi: 10.1001/jamanetworkopen.2023.17200 (PMC10248743; doi:10.1001/jamanetworkopen.2023.17200)
Supplement: Supplement 1. — eAppendix. PAINT Study Group eTable 1. Analgesics and Sedatives to Accompany Table 3 eTable 2. Analgesics and Sedatives to Accompany Table 4 [file jamanetwopen-e2317200-s001.pdf]

Supplemental Online Content

Poonai N, Cohen DM, MacDowell D, et al; Paediatric Emergency Research Networks (PERN) PAINT Study Group. Sedation and analgesia for reduction of pediatric ileocolic intussusception. *JAMA Netw Open*. 2023;6(6):e2317200. doi:10.1001/jamanetworkopen.2023.17200

eAppendix. PAINT Study Group
eTable 1. Analgesics and Sedatives to Accompany Table 3
eTable 2. Analgesics and Sedatives to Accompany Table 4

This supplemental material has been provided by the authors to give readers additional information about their work.

© 2023 Poonai N et al. *JAMA Network Open.*

Appendix 1. PAINT Study Group

Network<br>PECARN/PEMCRC	Institution	Site Investigator(s)
United States	University of Texas Southwestern Medical Center, Dallas, Texas	Nishit Patel, Marilyn Elliott
United States	Rady Childrens Specialist, Encinitas, California	Yvette Wang
United States	Children's Hospital Los Angeles, Los Angeles, California	Alan Nager, Sofronia (Sofie) Munoz Ringold
United States	Children's National Hospital, Washington, D.C.	Camilo Gutierrez
United States	Children's Hospital of Orange County, Orange, California	Theodore Heyming, Kellie Bacon
United States	Seattle Childrens Hospital, Seattle, Washington	Rebekah Burns, Eileen Klein, Indi Trehan
United States	Ann & Robert H. Lurie Children's Hospital of Chicago, Chicago, Illinois	Emily Roben
United States	Nationwide Childrens Hospital, Columbus, Ohio	Daniel Cohen, Doug MacDowell
United States	Cincinnati Children's Hospital Medical Center, Cincinnati, Ohio	Matthew J. Lipshaw
United States	Children’s Healthcare of Atlanta, Atlanta, Georgia	Carmen Sulton
United States	Boston Children's Hospital, Boston, Massachusetts	Joyce Li
United States	Texas Children's Hospital, Houston, Texas	Aderonke Ojo
United States	OU Health Sciences Center, Oklahoma City, Oklahoma	Cecilia Guthrie, Valorie Owens
United States	Washington University School of Medicine, St. Louis, Missouri	Kimberly S. Quayle
United States	University at Buffalo, Buffalo, New York	Heather Territo
United States	Nemours/Alfred I. duPont Hospital for Children, New Castle County, Delaware	Susan M. Kelly
United States	University of Michigan, Ann Arbor, Michigan	Vincent Cervantes, Alicia Rolin
United States	Children's Mercy Kansas City, Kansas City, Missouri	Shobhit Jain, Dan Kornfeld
United States	University of Mississippi Medical Center, Jackson, Mississippi	Justin Davis
United States	SUNY Upstate Medical University, Syracuse, New York	Matthew D. Thornton
United States	Norton Children's Hospital, Louisville, Kentucky	Kerry Caperell
United States	University of California, San Francisco, California	Jackie Grupp-Phelan, Margaret Lin-Martore
United States	University of Minnesota Masonic Children's Hospital, Minneapolis, Minnesota	Iluonose Amoni, Elizabeth Ramey, Ose Amoni
United States	Children's Hospital Colorado Anschutz Medical Campus, Aurora, Colorado	Anna Abrams
United States	SIU School of Medicine, Springfield, Illinois	Myto Duong, Sharon Kim
United States	Lincoln Medical Center, Bronx, New York	Dominic Brunello, Muhammad Waseem, Susan Wojcik
United States	John R. Oishei Children's Hospital, Buffalo, New York	Heather Territo
United States	Primary Children's Hospital, Salt Lake City, Utah	Matthew Steimle

© 2023 Poonai N et al. *JAMA Network Open.*

**PERC**		
Canada	The Hospital for Sick Children, Toronto, Ontario	Adrienne L. Davis
Canada	CHU Sainte-Justine, Montreal, Quebec	Jocelyn Gravel, Evelyne Doyon-Trottier
Canada	Alberta Children's Hospital, Calgary, Alberta	Neta Bar Am, Graham Thompson
Canada	British Columbia (BC) Children's Hospital, Vancouver, British Columbia	Vikram Sabhaney, Garth Meckler
Canada	Children's Hospital of Eastern Ontario, Ottawa, Ontario,	Rini Jain
Canada	Children's Hospital - London Health Sciences Centre, London, Ontario	Naveen Poonai
Canada	Stollery Children's Hospital, Edmonton, Alberta	Samina Ali
**REPEM**		
Italy	Agostino Gemelli University Policlinic, Rome	Danilo Buonsenso
Italy	University of Padova, Padova	Silvia Bressan, Tiziana Zangardi
Italy	Gaslini Children's Hospital, Genova	Giovanna Villa
Italy	University Hospital Meyer, Florence	Martina Giacalone, Idanna Sforzi
Switzerland	Children's Hospital Zurich - Eleonore Foundation, Zürich	Michelle Seiler
Switzerland	Hôpitaux Universitaires de Genève, Geneva	Cyril Sahyoun
Switzerland	University Hospital Bern, Bern	Fabrizio Romano
Hungary	Heim Pál Children's Hospital, Budapest	Zsolt Bognar, Szofia Hajos-Kalcakosz
Israel	Rambam Health Care Campus, Haifa	Eli Hershman
Israel	Schneider Children's Medical Center, Petah Tikva	Lisa Amir
Belgium	Ghent University Hospital, Ghent, Belgium	Said Hachimi-Idrissi
Latvia	Children's Clinical University Hospital, Riga	Zanda Pucuka, Astra Zviedre, Emīlija Zeltiņa, Jānis Kolbergs
**PREDICT**		
Australia	Queensland Children's Hospital, South Brisbane	Natalie Phillips
Australia	Monash Medical Centre, Clayton	Simon Craig
Australia	Perth Children's Hospital, Nedlands	Meredith Borland, Sharon O'Brien
Australia	Children's Hospital at Westmead, Sidney	Jeanette Marchant
Australia	Women's and Children's Hospital, North Adelaide	Amit Kochar, Gaby Nieva
Australia	Gold Coast University Hospital, Southport	Shane George
Australia	Sydney Children's Hospital, Randwick, Sidney	Victoria Pennington

© 2023 Poonai N et al. *JAMA Network Open.*

PERUKI		
United Kingdom	Bristol Royal Hospital for Children, Bristol	Sarah Sheedy, Mark Lyttle
United Kingdom	Royal Hospital for Sick Children, Edinburgh, Scotland	Jen Browning
United Kingdom	Royal Hospital for Children, Glasgow, Scotland	Steve Forester, Anna McLoughlin
United Kingdom	Birmingham Children's Hospital, Birmingham	Stuart Hartshorn
United Kingdom	Evelina London Children's Hospital, London	Chaman Urooj , Lucy Johnston
United Kingdom	Royal Alexandra Children's Hospital, Brighton	Emily Walton, Charlotte Harper
United Kingdom	Sheffield Children's Hospital, Sheffield	Liz Binham
United Kingdom	Leicester Royal Infirmary, Leicester	Deepika Subrahmanyam Puthucode
United Kingdom	John Radcliffe Hospital, Oxford	Phil Peacock
United Kingdom	Leeds General Infirmary, Leeds	James Conroy
**RISeuP/SPERG**		
Spain	Hospital Universitario Gregorio Marañon, Madrid	Rafa Marañon
Spain	Cruces University Hospital, Bilbao	Silvia Garcia
Spain	Consorci Corporació Sanitària Parc Taulí, Sabadell	Nuria Cahís
Spain	Hospital Donostia, San Sebastián	Amaia Cámara-Otegui
Spain	Hospital Universitario Joan XXIII., Tarragona	Arantxa Gomez
Spain	Hospital Virgen del Rocio, Sevilla	Maria J Carbonero
Spain	Hospital Francesc de Borja, Valencia	Carlos M Angelats-Romero
**RIDEPLA**		
Costa Rica	National Children's Hospital, San José	Adriana Yock-Corrales
Argentina	Hospital Garrahan, Buenos Aires	Gabriela Hualde
**OTHER**		
Laos	Lao Friends Hospital for Children, Laos	Indi Trehan
Switzerland	Reseau Hospitalier Neuchateloi, La Chaux-de-Fonds	Fabian Spigariol
Switzerland	Children's Hospital - Lucerne Cantonal Hospital, Lucerne	Alex Donas
Switzerland	Children's Hospital of Eastern Switzerland St.Gallen, St. Gallen	Cinthia Gübeli Linné
Italy	Policlinico of Milan*, Milan	Laura Dell'Era, Alessia Rocchi
Italy	Filippo Del Ponte Hospital*, Varese	Alessia Pedrazzini
Italy	Hospital Burlo Garofolo*, Trieste	Giorgio Cozzi, Dino Barbi
Italy	Hospital for Women and Children*, Verona	Laura Baggio, Giovana Fauci
Italy	Vito Fazzi Hospital*, Lecce	Assunta Tornesello
Italy	Santobono-Pausilipon Children's Hospital*, Naples	Angela Mauro

Notes:
PECARN=Pediatric Emergency Care Applied Research Network. PEMCRC=Pediatric Emergency Medicine Collaborative Research Committee. PERC=Pediatric Emergency Research Canada. REPEM=Research in European Pediatric Emergency Medicine. PREDICT=Paediatric Research in Emergency Departments International Collaborative in Australia and New Zealand. PERUKI=Paediatric Emergency Research in the UK and Ireland. RISeuP/SPERG=Research Network of the Spanish Society of Pediatric Emergency/Spanish Pediatric Emergency Medicine Research Group. RIDEPLA= Red de Investigacion y Desarrollo de la Emergencia Pediatrica Latinoamericana (Research and Development of Pediatric Emergency Medicine in Latin America).
*Affiliated to the PIPER (Pain in Paediatric Emergency Room), an Italian research network.

© 2023 Poonai N et al. *JAMA Network Open.*

eTable 1. Analgesics and Sedatives to Accompany Table 3

Unadjusted and Adjusted Analyses of Variables Associated with Perforation During Reduction of Ileocolic Intussusception

Drug	No. (%)
**Analgesic**	**N=466**
morphine (alone)	276 (59.2)
(fentanyl (alone)	73 (15.7)
acetaminophen/paracetamol (alone)	40 (8.6)
ibuprofen (alone)	20 (4.3)
acetaminophen/paracetamol + morphine nalbuphin (alone)	16 (3.4)
acetaminophen/paracetamol + fentanyl metamizole/dipyrone (alone)	11 2.4
ibuprofen + acetaminophen/paracetamol	7 (1.5)
morphine + fentanyl	6 (1.3)
oxycodone (alone)	5 (1.1)
unspecified analgesia	3 (0.6)
ibuprofen + morphine	1 (0.2)
ibuprofen + fentanyl	1 (0.2)
acetaminophen/paracetamol + nalbuphine	1 (0.2)
metamizole/dipyrone + fentanyl	1 (0.2)
ibuprofen + acetaminophen/paracetamol + morphine	1 (0.2)
ibuprofen + acetaminophen/paracetamol + fentanyl	1 (0.2)
acetaminophen/paracetamol + morphine + fentanyl	1 (0.2)
**Sedative**	**N=334**
midazolam (alone)	168 (50.3)
midazolam + ketamine	51 (15.3)
unspecified sedative (alone)	42 (12.6)
propofol (alone)	11 (3.3)
midazolam + propofol	9 (2.7)
ketamine (alone)	8 (2.4)
propofol + fentanyl + sevoflurane + mivacron	6 (1.8)
ketamine + propofol	5 (1.5)
propofol + fentanyl + sevoflurane	5 (1.5)
midazolam + fentanyl	4 (1.2)
midazolam + ketamine + propofol	4 (1.2)
midazolam + propofol + fentanyl	4 (1.2)
propofol + fentanyl + sevoflurane + lystenon	3 (0.9)
midazolam + thiopental	2 (0.6)
sevoflurane (alone)	1 (0.3)
midazolam + sevoflurane	1 (0.3)
ketamine + fentanyl	1 (0.3)
propofol + fentanyl	1 (0.3)
propofol + sevoflurane	1 (0.3)
fentanyl + sevoflurane + tracrium	1 (0.3)
propofol + sevoflurane + succinylcholine	1 (0.3)
ketamine + fentanyl + sevoflurane + mivacron	1 (0.3)
propofol + fentanyl + lystenon	1 (0.3)
propofol + fentanyl + mivacron	1 (0.3)
propofol + fentanyl + sevoflurane + tracrium	1 (0.3)
propofol + sevoflurane + mivacron + remifentanil	1 (0.3)

© 2023 Poonai N et al. *JAMA Network Open.*

eTable 2. Analgesics and Sedatives to Accompany Table 4

Unadjusted and adjusted analyses of variables associated with failed reduction[a] of ileocolic intussusception

Drug	No. (%)
**Analgesic**	**N=464**
morphine (alone)	275 (59.3)
fentanyl (alone)	73 (15.7)
acetaminophen/paracetamol (alone)	40 (8.6)
ibuprofen (alone)	20. (4.3)
acetaminophen/paracetamol + morphine	16 (3.4)
nalbuphine (alone)	11 (2.4)
acetaminophen/paracetamol + fentanyl	7 (1.5)
metamizole/dipyrone (alone)	5 (1.1)
ibuprofen + acetaminophen/paracetamol	5 (1.1)
morphine + fentanyl	3 (0.6)
oxycodone (alone)	1 (0.2)
unspecified analgesia	1 (0.2)
ibuprofen + morphine	1 (0.2)
ibuprofen + fentanyl	1 (0.2)
acetaminophen/paracetamol + nalbuphine	1 (0.2)
metamizole/dipyrone + fentanyl	1 (0.2)
ibuprofen + acetaminophen/paracetamol + morphine	1 (0.2)
ibuprofen + acetaminophen/paracetamol + fentanyl	1 (0.2)
acetaminophen/paracetamol + morphine + fentanyl	1 (0.2)
**Sedative**	**N=333**
midazolam (alone)	168 (50.5)
midazolam + ketamine	51 (15.3)
unspecified sedative (alone)	41 (12.3)
propofol (alone)	11 (3.3)
midazolam + propofol	9 (2.7)
ketamine (alone)	8 (2.4)
propofol + fentanyl + sevoflurane + mivacron	6 (1.8)
ketamine + propofol	5 (1.5)
propofol + fentanyl + sevoflurane	5 (333)
midazolam + fentanyl	4 (1.2)
midazolam + ketamine + propofol	4 (1.2)
midazolam + propofol + fentanyl	4 (1.2)
propofol + fentanyl + sevoflurane + lystenon	3 (0.9)
midazolam + thiopental	2 (0.6)
sevoflurane (alone)	1 (0.3)
midazolam + sevoflurane	1 (0.3)
ketamine + fentanyl	1 (0.3)
propofol + fentanyl	1 (0.3)
propofol + sevoflurane	1 (0.3)
fentanyl + sevoflurane + tracrium	1 (0.3)
propofol + fentanyl + lystenon	1 (0.3)
propofol + fentanyl + mivacron	1 (0.3)
propofol + sevoflurane + succinylcholine	1 (0.3)
ketamine + fentanyl + sevoflurane + mivacron	1 (0.3)
propofol + fentanyl + sevoflurane + tracrium	1 (0.3)
propofol + sevoflurane + mivacron + remifentanil	1 (0.3)

© 2023 Poonai N et al. *JAMA Network Open.*
