# Supplementary material for: Sedation and Analgesia for Reduction of Pediatric Ileocolic Intussusception
Source: JAMA Netw Open. 2023 Jun 7;6(6):e2317200. doi: 10.1001/jamanetworkopen.2023.17200 (PMC10248743; doi:10.1001/jamanetworkopen.2023.17200)
Supplement: Supplement 2. — Nonauthor Collaborators [file jamanetwopen-e2317200-s002.pdf]

Supplemental Online Content: Nonauthor Collaborators
*First name, last name, and suffix (if applicable) are required and will appear in PubMed.

***Group Name(s): PECARN/PEMCRC, REPEM, PREDICT, PERUKI**							
***First Name and Middle Initial(s)**	***Last Name**	***Suffix (eg, Jr, III)**	Academic Degrees	Institution	Location (city, state/province, country)	Role or Contribution, eg, chair, principal investigator	Group (if more than 1 Group listed in the byline) and/or Subgroup (eg, Steering Committee)
Nishit	Patel			University of Texas Southwestern Medical Center, Dallas, Texas			PECARN/PEMCRC
Camilo	Gutierrez			Children's National Hospital, Washington, D.C.			PECARN/PEMCRC
Emily	Roben			Ann & Robert H. Lurie Children's Hospital of Chicago, Chicago, Illinois			PECARN/PEMCRC
Chris	Pruitt			Medical University of South Carolina			PECARN/PEMCRC
Kimberly S.	Quayle			Washington University School of Medicine, St. Louis, Missouri			PECARN/PEMCRC
Alicia	Rolin			University of Michigan, Ann Arbor, Michigan			PECARN/PEMCRC
Dan	Kornfeld			Children's Mercy Kansas City, Kansas City, Missouri			PECARN/PEMCRC
Justin	Davis			University of Mississippi Medical Center, Jackson, Mississippi			PECARN/PEMCRC
Jackie	Grupp-Phelan			University of California, San Francisco, California			PECARN/PEMCRC
Amanda	Bogie			OU Health Sciences Center, Oklahoma City, Oklahoma			PECARN/PEMCRC
Heather	Territo			John R. Oishei Children's Hospital, Buffalo, New York			PECARN/PEMCRC
Eli	Hershman			Rambam Health Care Campus, Haifa			REPEM
Jānis	Kolbergs			Children's Clinical University Hospital, Riga			REPEM
Virginia	Stanton			Children’s Hospital at Westmead, Sidney			PREDICT
Sarah	Sheedy			Bristol Royal Hospital for Children, Bristol			PERUKI
Steve	Forester			Royal Hospital for Children, Glasgow, Scotland			PERUKI
Liz	Binham			Sheffield Children's Hospital, Sheffield			PERUKI
Laura	Dell’Era			Policlinico of Milan, Milan			
Assunta	Tornesello			Vito Fazzi Hospital, Lecce			
