# Supplementary material for: Sedation and Analgesia for Reduction of Pediatric Ileocolic Intussusception
Source: JAMA Netw Open. 2023 Jun 7;6(6):e2317200. doi: 10.1001/jamanetworkopen.2023.17200 (PMC10248743; doi:10.1001/jamanetworkopen.2023.17200)
Supplement: Supplement 3. — Data Sharing Statement [file jamanetwopen-e2317200-s003.pdf]

Data Sharing Statement

Poonai. Sedation and Analgesia for Reduction of Pediatric Ileocolic Intussusception. *JAMA Netw Open*. Published June 07, 2023. doi:10.1001/jamanetworkopen.2023.17200

Data
Data available: Yes
Data types: Deidentified participant data
How to access data: De-identified participant data will be made available with publication after approval of a proposal with a signed data sharing agreement and upon request of the corresponding author.
When available: With publication

Supporting Documents
Document types: None

Additional Information
Who can access the data: Researchers whose proposed use of the data has been approved
Types of analyses: Any specified purpose
Mechanisms of data availability: With signed data access agreement
Any additional restrictions: None
